# The anti-inflammatory astrocyte revealed: the role of the microbiome in shaping brain defences

**DOI:** 10.1038/s41392-021-00577-5

**Published:** 2021-04-10

**Authors:** Alexei Verkhratsky, Peter Illes, Yong Tang, Alexey Semyanov

**Affiliations:** 1 Faculty of Biology, Medicine and Health, The University of Manchester, Manchester, UK; 2 Achucarro Center for Neuroscience, IKERBASQUE, Basque Foundation for Science, 48011 Bilbao, Spain & Department of Neurosciences, University of the Basque Country UPV/EHU and CIBERNED, Leioa, Spain; 3 Sechenov First Moscow State Medical University, Moscow, Russia; 4 School of Acupuncture and Tuina and International Collaborative Centre on Big Science Plan for Purinergic Signalling, Chengdu University of Traditional Chinese Medicine, Chengdu, China; 5 Rudolf Boehm Institute for Pharmacology and Toxicology, University of Leipzig, Leipzig, Germany; 6 Shemyakin-Ovchinnikov Institute of Bioorganic Chemistry, Russian Academy of Sciences, Moscow, 117997 Russia; 7 Faculty of Biology, Moscow State University, Moscow, Russia

**Keywords:** Cellular neuroscience, Ageing

The paper, published recently in Nature by the group of Francisco Quintana,^1^ describes the anti-inflammatory astrocytes, activity of which is tuned by the microbiotome and meningeal natural killer (NK) cells.

Astrocytes are a sub-type of neuroglia responsible for homoeostasis and defence of the nervous system. Pathological reactions of neuroglial cells in various neurological disorders have been identified and characterised in the end of the 19th century; in particular hypertrophy of astrocytes was recognised as a frequent morbid change accompanying diseases of the central nervous system (CNS). Subsequently, hyperplasia and proliferation of astrocytes in response to polyetiological insults to the nervous tissue were formalised in a concept of reactive astrogliosis, which was considered to be a universal sign of neuropathology, a kind of stereotypic response that provided “splint” (as it was called by Wilder Penfield) to the nervous tissue and filled the spaces left by disintegrating neurons; the ultimate outcome of astrogliosis was a formation of a glial scar.^2^ This view has dominated neurological thoughts for almost a century; however, recent two decades led to a fundamental revision of principles of astrogliopathology.^3,4^ First, it has been found that various diseases including, for instance, neuropsychiatric disease or various leucomalacia are associated with or even driven by astroglial asthenia, atrophy or loss of function. Second, it appeared that astrogliosis generates multiple reactive phenotypes, characterised by distinct gene expression signatures and functional outcomes^3,5^; reactive astrocytes often act as neuroprotectors but sometimes acquire deleterious features. Inhibition of reactive astrogliosis may either exacerbate or alleviate neurological outcome. Various reactive or asthenic astrocytic phenotypes may develop sequentially or simultaneously in different brain regions under the same pathological context. Otherwise, distinct pathological forms of astrocytes can be linked to the disease stage, reflecting a high degree of pathological plasticity of astroglia. Astrocytic phenotypes and functions are also affected by ageing, while the remodelling of astrocytes in the old CNS can define the susceptibility of the nervous tissue to age-dependent neurodegenerative diseases.

A previously unknown population of anti-inflammatory astrocytes has been identified and characterised in the mouse brain by a combination of state-of-the-art techniques including single-cell RNA sequencing, high-throughput flow cytometry screening, and CRISPR–Cas9-based genetic manipulations.^1^ These specialised astrocytes express the lysosomal associated protein 1 (LAMP1) and the death receptor activator TRAIL (encoded by *Tnfsf10 gene)*, which both confer anti-inflammatory capabilities. The TRAIL stands for tumour necrosis factor-related apoptosis-inducing ligand; it belongs to the family of tumour necrosis factors, which instigate apoptosis through engaging death receptors and their downstream molecular cascades. The TRAIL-positive astrocytes limit neuroinflammation in the experimental autoimmune encephalomyelitis (EAE) by initiating apoptotic death of infiltrating T-lymphocytes. Specific inactivation of TRAIL in astrocytes by inactivating the *Tnfsf10 gene* with *Gfap*-driven CRISPR–Cas9 lentivirus aggravated EAE progression. The anti-inflammatory TRAIL astrocytes seem to populate the spinal cord; they were not identified in the cortex and cerebellum.

**Fig. 1:**
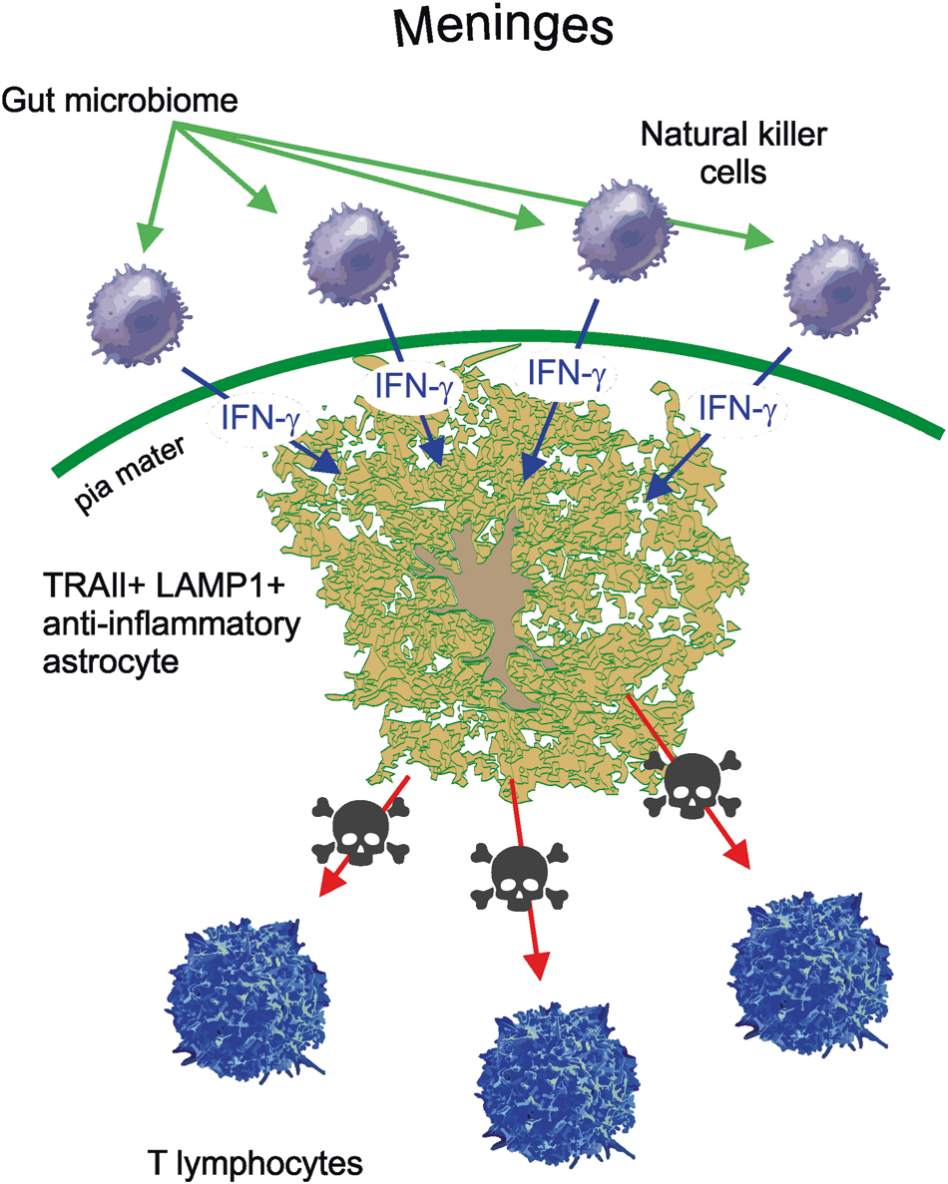
Anti-inflammatory astrocytes. The subpopulation of anti-inflammatory astrocytes is present in the spinal cord of mice and possibly of humans.^5^ These astrocytes specifically express the lysosomal associated protein 1 (LAMP1) and the death receptor ligand TRAIL; the latter after being released activate death receptors on T lymphocytes instigating their apoptosis. Expression of TRAIL in astrocytes is controlled by IFN-γ secreted by meningeal natural killer cells; IFN-γ reaches astrocytic endfeet through subarachnoid cerebrospinal fluid. The IFN-γ natural killer cells are, in their turn, under control from systemic factors including the gut microbiome; depletion of the latter substantially reduces the number of IFN-γ secreting natural killer cells

The TRAIL-positive astrocytes are located close to meninges in mice and in humans; notably, their population is decreased in tissues obtained from patients with diagnosed multiple sclerosis. The anti-inflammatory astrocytes are regulated by NK cells residing in meninges (Fig. 1); this regulation is mediated by interferon-γ (IFN-γ). The NK cells are a part of innate tissue immunity; these cells are large granular lymphocytes that are swiftly recruited in response to insults. The NK cells are cytolytic and secrete various cytokines; these cells are known to limit neuroinflammation, particularly in the context of multiple sclerosis. The NK cells are heterogeneous; a sub-population of them produce and secrete IFN-γ, a relatively small molecule (m.w. ~16.8 kDa), which can diffuse through subarachnoid cerebrospinal fluid and reach astrocytic endfeet. When perceived by astrocytic receptors, IFN-γ launches the yet unknown signalling cascade that upregulates TRAIL expression and hence stimulates anti-inflammatory astrocyte defence. The state of NK cells is modulated by systemic factors, and in particular, the production of IFN-γ depends on the gut flora. Depletion of the microbiome by treatment with antibiotics resulted in a substantial decrease of the IFN-γ producing meningeal NK cells, although their total number remains unchanged. Such a reduction translates into downregulation of the anti-inflammatory astrocytes, thus presumably limiting their protective potential.

Identification of a specialised sub-type of anti-inflammatory astrocytes further widens the repertoire or astrocytic capabilities to restrain diseases and adds a new dimension to our understanding of astroglial plasticity in the healthy and diseased brain. The variations in the state of anti-inflammatory astrocytes are regulated by NK meningeal cells, which, in their turn, are controlled by systemic immunity and in particular, by the gut microbiome. The gut—NK cell—astrocytes axis clearly demonstrates the fundamental role of systemic factors in the regulation of various aspects of CNS function. The status of the gut–brain axis also defines the vulnerability of the nervous tissue to multiple diseases, as indeed deficiency in anti-inflammatory astrocytes may be directly linked to neuroinflammatory disorders such as multiple sclerosis. Manipulation with the gut microbiome may, therefore, represent a potentially powerful therapeutic strategy. It also remains to be seen whether anti-inflammatory astrocytes operate only in the spinal cord or similar cells populate other brain parts; it is also imperative to discern morphological and physiological peculiarities of these astrocytes.

Conceptually, the state of neuroglia defines the brain vulnerability to pathology. In this respect, all types of neuroglia seem to work in concert to keep the homoeostasis of the nervous tissue intact in the face of ongoing environmental challenges. Insults to the brain similarly trigger a concerted response of all neuroglial cells aimed at resolution of pathology, regeneration and return to the physiological homoeostatic state. Neuroglial failure at every stage facilitates neuropathology, whereas deficient defence promotes the death of neural tissue. Neuroglia, therefore, represents a primary target for preventive medicine as manipulation with protective glial phenotypes may arrest or retard pathological evolution. The discovery of a new set of astroglia not only adds to astroglial heterogeneity and plasticity but also hints at many more subtypes of astrocytes operational in healthy and pathological nervous tissue.
